# Microbiological and clinical predictors of sepsis-associated encephalopathy in bloodstream infections: a retrospective cohort study

**DOI:** 10.3389/fcimb.2025.1548370

**Published:** 2025-03-07

**Authors:** Yunhan Fei, Zhaowei Hao, Xinwei Zheng, Xiang Ji, Wenjuan Zhao

**Affiliations:** Department of Emergency, Tianjin Huanhu Hospital, Tianjin, China

**Keywords:** sepsis-associated encephalopathy (SAE), coagulase-negative staphylococci, microbial risk factors, in-hospital mortality, cohort study

## Abstract

**Background:**

Sepsis-associated encephalopathy (SAE) is a common and severe complication of sepsis, significantly contributing to morbidity and mortality. The impact of specific blood-borne pathogens on SAE risk and prognosis remains unclear. This study investigates the microbiological and clinical factors associated with bloodstream infection-induced SAE.

**Methods:**

We analyzed data from the MIMIC-IV database, including 16,141 sepsis patients who met inclusion criteria. Patients were divided into SAE and non-SAE groups for comparison. Multivariate regression identified independent risk factors for SAE and associated outcomes, including in-hospital mortality.

**Results:**

Coagulase-negative staphylococci (CoNS) was identified as a key microbial risk factor for SAE (HR=1.919, P<0.001), though it was not associated with in-hospital mortality. Higher SOFA scores, mechanical ventilation, and prolonged antibiotic use significantly increased SAE risk. Laboratory tests revealed higher white blood cell counts, platelet levels, and metabolic abnormalities in SAE patients. Methicillin-resistant Staphylococcus aureus (MRSA) was linked to increased mortality in SAE patients (HR=3.423, P<0.001).

**Conclusion:**

Coagulase-negative staphylococci is a significant risk factor for SAE development, but not for mortality. Advanced age, female gender, higher SOFA scores, and mechanical ventilation further contribute to SAE risk. Early identification and targeted management of pathogens, particularly methicillin-resistant Staphylococcus aureus, are crucial for improving SAE outcomes.

## 1 Introduction

Sepsis remains the leading cause of death among critically ill patients (Singer et al., 2016), with an estimated 48.9 million cases and 11 million sepsis-related deaths occurring annually worldwide (GBD 2021 Nervous System Disorders Collaborators, 2024). Sepsis-associated encephalopathy (SAE), a common neurological complication, affects up to 70% of sepsis patients (Pandharipande et al., 2013), and represents a significant contributor to both acute and long-term mortality, as well as cognitive impairment (Eidelman et al., 1996; Annane and Sharshar, 2015). While systemic infection and inflammation are well-established contributors to SAE, the specific roles of various pathogens in influencing SAE risk and prognosis remain poorly understood. Current research has predominantly focused on common sepsis pathogens, such as Staphylococcus aureus and Escherichia coli (Zhang et al., 2012; Zhao et al., 2021; Sokołowska et al., 2024). However, a comprehensive analysis of the differential impact of these pathogens on SAE development is lacking, limiting the advancement of targeted preventive and therapeutic strategies for high-risk pathogens.

Early identification and timely management of brain injury are critical for improving the survival and prognosis of sepsis patients. While previous studies have largely explored the influence of infection sites on SAE development (Gofton and Young, 2012), relatively little attention has been paid to the relationship between specific microorganisms and SAE. Bloodstream infections are a key source of systemic infections in sepsis, and distinct pathogens may contribute to SAE onset and outcomes through unique mechanisms of infection and virulence. Pathogens regulate the production of virulence factors and metabolites, enabling them to breach the blood-brain barrier and induce neurological damage (Herold et al., 2019; Yamada and Kielian, 2019). For instance, bacterial species like Staphylococcus aureus utilize mechanisms such as biofilm formation and antibiotic resistance to enhance their pathogenicity (Schilcher and Horswill, 2020). These observations underscore the need for a systematic evaluation of the roles that different blood-borne pathogens play in SAE risk and prognosis.

This study aims to investigates the microbiological and clinical factors associated with bloodstream infection-induced SAE. By analyzing the infection characteristics and patient prognoses associated with distinct pathogens, we seek to identify high-risk strains that pose the greatest threat of SAE and are linked to poorer outcomes.

## 2 Methods

### 2.1 Data source

The data for this study was sourced from the Critical Care Medical Information Market (MIMIC-IV, version 3.0). Information was extracted for patients admitted to the BIDMC emergency department or one of the intensive care units (ICUs) between 2008-2022. A master patient list was compiled, containing all medical record numbers corresponding to patients admitted to the ICU or emergency department during this period. The database includes various clinical records, such as demographic information, vital signs, microbiological events, drug prescriptions, laboratory test results, and other charted events.

This study specifically focused on blood culture results from sepsis patients and analyzed 12 major bacterial strains identified as blood culture positive. Additionally, the International Classification of Diseases, Ninth Edition (ICD-9) codes, recorded by hospital staff at the time of patient discharge, were utilized to classify diagnoses.

The author of this study has successfully completed the Collaborative Institutional Training Initiative (CITI) examination (certification number: 58218785), which grants authorized access to the MIMIC-IV database. Raw data was extracted using Structured Query Language (SQL) via Navicat software and subsequently processed and analyzed using R software.

### 2.2 Patient population

Inclusion criteria were as follows: (1) Patients with sepsis 3.0 (Singer et al., 2016). (2) age≥18 years old. (3) 24h ≤time stay in the ICU ≤30 days. (4) SOFA score ≥ 2. To ensure the accuracy of the results, this study excluded cases that may interfere with the diagnosis of septic encephalopathy: (Sonneville et al., 2017; Yang et al., 2020; Li et al., 2022): 1) patients with brain injury (e.g., traumatic brain injury, meningitis, encephalitis intracerebral hemorrhage, cerebral embolism, ischemic stroke, epilepsy, brain tumor or intracranial infection, and any other cerebrovascular disease); 2) mental disorders and neurological disease; 3) chronic alcohol or drug abuse; 4) metabolic encephalopathy, hepatic encephalopathy, hypertensive encephalopathy, hypoglycemic coma, and other liver disease or kidney disease that affected consciousness; 5) severe electrolyte imbalances or glycemic disturbances, including hyponatremia (<120 mmol/l), hyperglycemia (>180 mg/dl), or hypoglycemia (<54 mg/dl); 6) those without GCS assessment; 7) Hyponatremia, hyperglycemia, and hypoglycemia can cause metabolic encephalopathy. Patients with any of the above-mentioned conditions were excluded.

Sepsis was defined as an infected patient on discharge according to ICD-9 codes and microbial culture positive. Sepsis-associated encephalopathy was defined as (1) patients with GCS < 15 (before sedation or surgery). (2) The patient was diagnosed with delirium, cognitive impairment, altered mental status according to the ICD-9 code. (3) The patient was treated with haloperidol during hospitalization. (4) Exclude consciousness disorders caused by other reasons. GCS has been identified as a clinically effective tool for characterizing SAEs and distinguishing them from sepsis (Sonneville et al., 2017). Extracting sedation or preoperative evaluation of GCS can help eliminate mental disorders or cognitive impairments caused by the use of sedatives and analgesics.

### 2.3 Statistical analysis

Sepsis patients with medical diagnostic information (n=16141) in the MIMIC database were included in the analysis. To ensure the validity of the results, the data was divided into Train dataset and Test dataset based on the enrollment time of participants: 70% of the participants are used as the training dataset (n=11296), and 30% of the participants (n=4845) are used as the testing dataset for model validation. (**Supplementary Table 1**).

The Shapiro Wilk test was used to detect distributions of data. This study uses continuous variables with non-normal distributions. Several continuous variables are described by the median and inter quartile range (IQR). There are also categorical variables that are expressed as a count and a percentage. The two groups of continuous variables were compared using a non-parametric test. The categorical variables were compared using Fisher’s exact test. To reduce multicollinearity between variables, we use a linear regression model to determine whether there is collinearity between independent variables. The VIF greater than 10 indicates that the model suffers from severe multicollinearity. Select variables based on literature review and clinical experience.

## 3 Results

### 3.1 Baseline demographic characteristics

**Figure 1:**
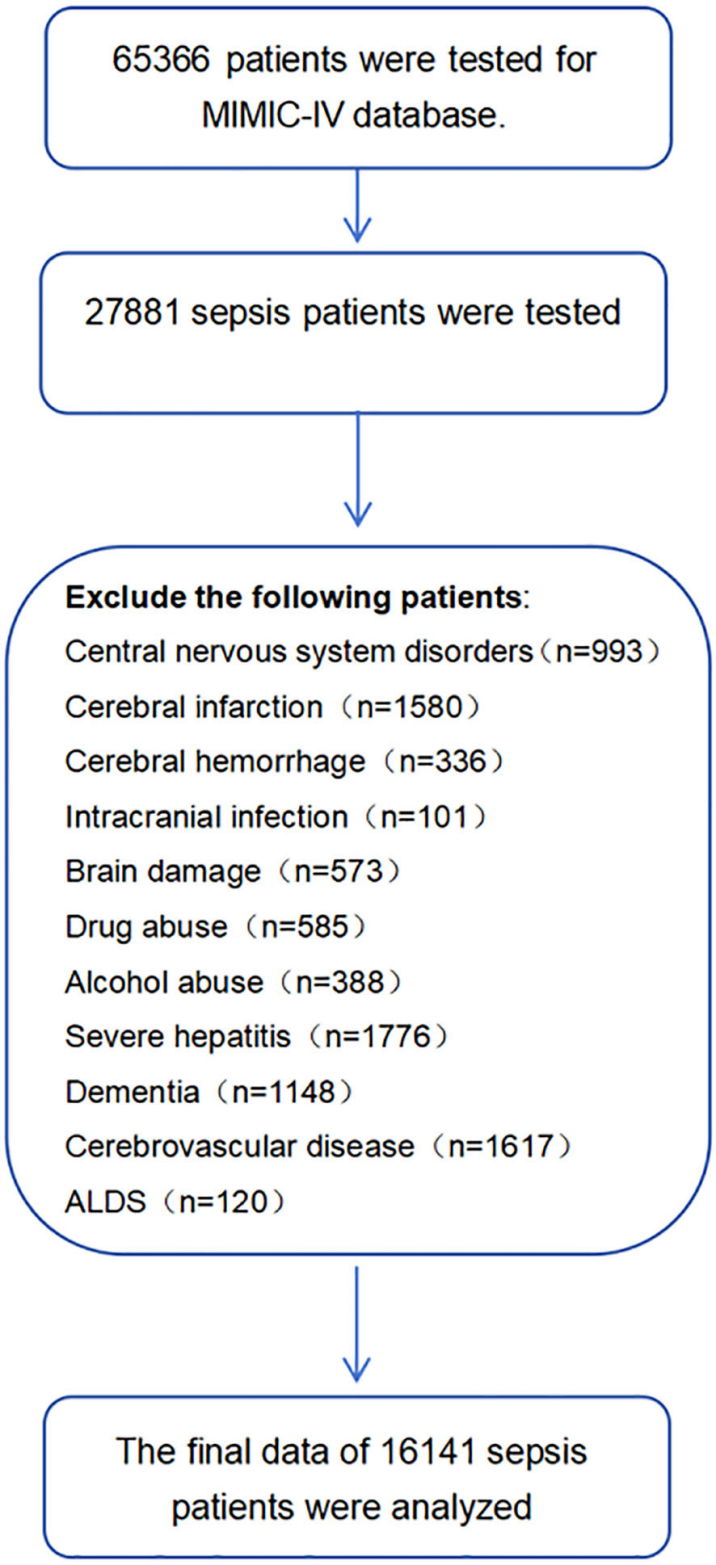
Flowchart of included participants from the MlMlC-lV.

After applying inclusion and exclusion criteria, 16141 sepsis patients were identified from the MIMIC database. The recruitment process is shown in **Figure 1**. The mean age of patients in the SAE group was 67.3 years, while the mean age of patients in the non SAE group was 67.1 years (P<0.001). In terms of gender ratio, females comprised 43.0% in the SAE group, which was higher than the 38.6% in the non SAE group (P<0.001). In addition, the Charlson score significantly increased in the SAE group (median 5 vs. 4, P<0.001). Distinct microbiological profiles were observed between SAE and non-SAE patients, particularly regarding coagulase-negative staphylococci (CoNS). The prevalence of this pathogen was significantly higher in the SAE group than in the non-SAE group (2.4% vs. 1.0%, P<0.001). Additionally, other pathogens such as Staphylococcus aureus (1.1% vs. 0.6%, P<0.001) and Staphylococcus epidermidis (0.4% vs. 0.2%, P<0.001) were more frequently detected in SAE patients. These results highlight the potential role of specific microbiological types, particularly within the Staphylococcus genus, in SAE pathogenesis. In laboratory tests, the white blood cell count and platelet levels of patients in the SAE group were significantly increased (white blood cell count: 11.34 vs. 10.79 × 10 ^ 9/L, P<0.001; Platelets: 184.85 vs. 169.96 × 10 ^ 9/L, P<0.001). In addition, the anion gap and maximum glucose level were also higher in the SAE group than in the non SAE group (anion gap: 16.13 vs. 15.54, P<0.001; Maximum glucose: 165.08 vs. 159.14, P<0.001), suggesting that patients in the SAE group may have more severe metabolic abnormalities. In terms of organ replacement therapy, the proportion of patients in the SAE group receiving mechanical ventilation and renal replacement therapy was significantly higher than that in the non SAE group (mechanical ventilation: 64.6% vs. 55.2%, P<0.001; Renal replacement therapy: 6.2% vs. 3.2%, P<0.001) (**Table 1**).

**Table 1:** Baseline characteristics and outcome of patient with sepsis.

Category	No-SAE	SAE	Total	*P* value
Age, mean (SD)	67.1 (15.1)	67.3 (16.4)	66.9 (16.0)	<0.001
Gender,n (%)				<0.001
Male	3083 (61.4)	6342 (57.0)	9425 (58.4)	
Female	1936 (38.6)	4780 (43.0)	6716 (41.6)	
Coexisting illness , n (%)				
Charlson	4 (2-6)	5 (3-7)	4 (3-7)	<0.001
Hypertension	2162 (43.1)	4720 (42.4)	6882 (42.6)	0.448
Diabetes	1453 (28.9)	3151 (28.3)	4604 (28.5)	0.420
Respiration	1313 (26.2)	3017 (27.1)	4330 (26.8)	0.200
Cardiovascular cerebro	13 (0.3)	80 (0.7)	93 (0.6)	<0.001
Renal	1072 (21.4)	2346 (21.1)	3418 (21.2)	0.702
Microbiology type, n (%)				<0.001
Culture-negative	4840 (96.4)	10461 (94.1)	15301 (94.8)	
Staphylococcus aureus	30 (0.6)	119 (1.1)	149 (0.9)	
Staphylococcus epidermidis	11 (0.2)	39 (0.4)	50 (0.3)	
Thrombin negative Staphylococcus aureus	52 (1.0)	266 (2.4)	318 (2.0)	
streptococcus	2 (0.04)	1 (0.01)	3 (0.02)	
Escherichia coli	34 (0.7)	83 (0.7)	117 (0.7)	
klebsiella pneumoniae	13 (0.3)	36 (0.3)	49 (0.3)	
Anaerobic bacteria such as fragile pseudomonas	8 (0.2)	19 (0.2)	27 (0.2)	
Candida albicans	6 (0.1)	8 (0.1)	14 (0.1)	
Grass green streptococcus	6 (0.1)	30 (0.3)	36 (0.2)	
Methicillin resistant golden grape balls	1 (0.02)	16 (0.14)	17 (0.11)	
Clostridium difficile	2 (0.03)	0 (0)	2 (0.01)	
Mixed infection	141 (0.3)	44 (0.4)	58 (0.4)	
Laboratory Examination				
hematocrit (IU/L)-min	29.30 (5.99)	29.83 (6.41)		<0.001
Hemoglobin -min	9.75 (2.01)	9.87 (2.13)		0.001
Platelets (g/dL)-min	169.96 (94.02)	184.85 (104.65)		<0.001
White blood cell ( × 10^∧9^/L)-min	10.79 (7.66)	11.34 (8.63)		<0.001
Aniongap-max	15.54 (4.92)	16.13 (4.88)		<0.001
Bicarbonate-max	21.33 (4.48)	21.35 (4.95)		0.001
BUN-max	27.83 (21.8)	30.62 (24.47)		<0.001
Creatinine-max	1.62 (1.74)	1.64 (1.67)		<0.001
glucose_max	159.14 (71.99)	165.08 (73.68)		<0.001
abs-Neutrophils-max (%)	11.31 (8.16)	11.81 (7.84)		0.003
sodium_max	139.25 (3.77)	139.85 (4.44)		<0.001
inr_min	1.35 (0.6)	1.35 (0.56)		0.512
Organ replacement therapy , n (%)				
Mechanical ventilation	2772 (55.2)	7187 (64.6)	9959 (61.7)	<0.001
Renal replacement therapy	161 (3.2)	694 (6.2)	855 (5.3)	<0.001
Score system, median (IQR)				
SAPS II	35.0 (28.0-42.0)	40.0 (31.0-50.0)	38.0 (30.0-47.0)	<0.001
SOFA	4.0 (3.0-6.0)	6.0 (4.0-8.0)	5.0 (3.0-7.0)	<0.001
GCS	15.0 (15.0-15.0)	13.0 (8.0-14.0)	14.0 (10.0-15.0)	<0.001
Use of vasopressors [n (%)]	2663 (53.1)	5817 (52.1)	8480 (52.5)	0.373
Length of admission to antibiotic use, days, mean (SD)	1.40 (3.32)	2.05 (4.35)	1.85 (4.07)	<0.001

### 3.2 Microbiology type and other key risk factors for SAE

**Figure 2:**
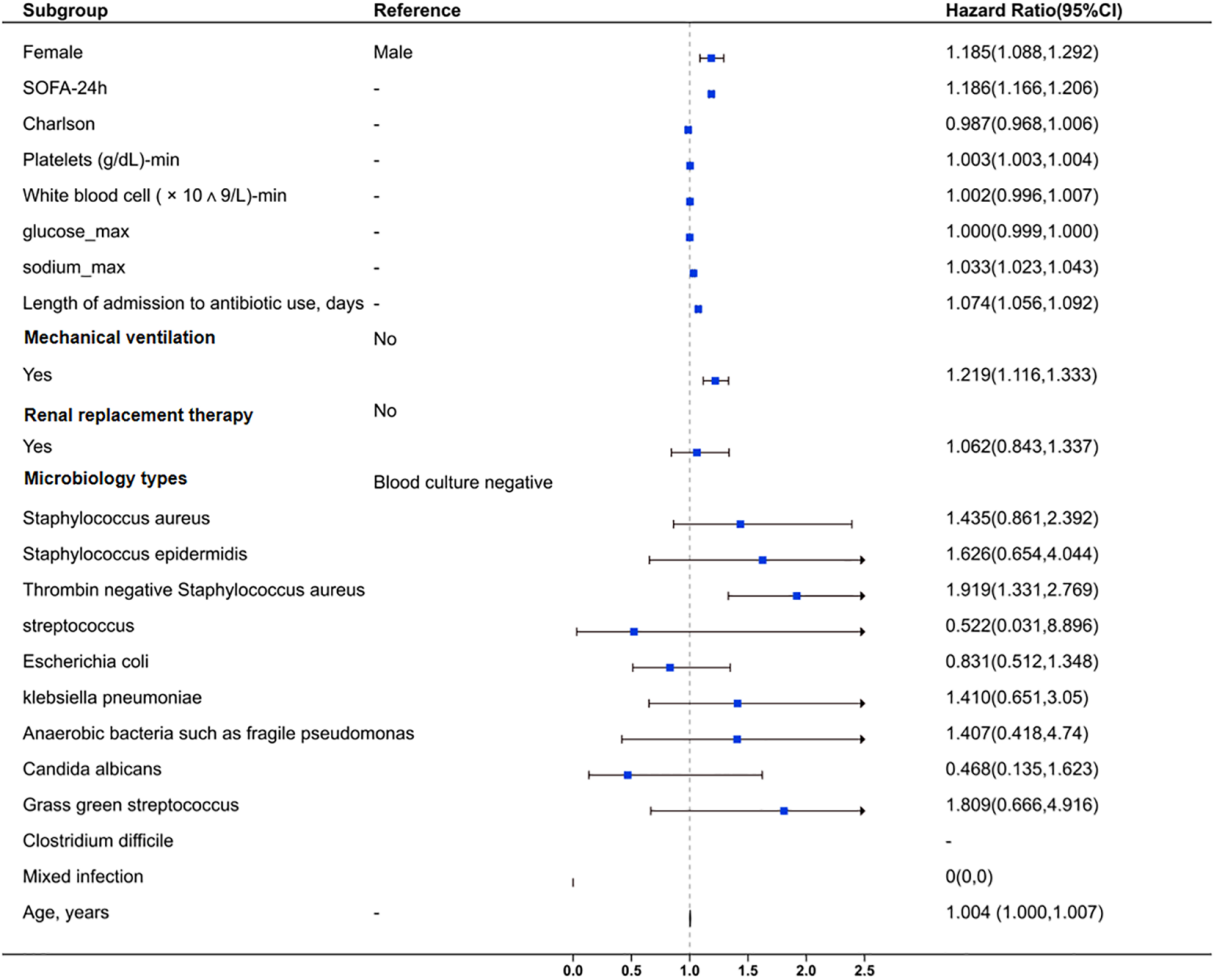
Forest plot for clinical and microbiological factors associated with sepsis-associated encephalopathy. The plot also evaluates the impact of mixed infections and age. Squares represent HR values, and horizontal lines indicate 95% CI. An HR > 1 suggests increased risk, while HR < 1 suggests reduced risk. The vertical dashed line marks HR = 1 (no effect).

Multivariate logistic regression analysis identified coagulase-negative staphylococci as an independent risk factor for SAE (HR=1.919, 95% CI: 1.331–2.769, P<0.001), alongside SOFA score (HR=1.186, 95% CI: 1.166–1.206, P<0.001) and mechanical ventilation requirement (HR=1.219, 95% CI: 1.116–1.333, P<0.001). This suggests that coagulase-negative staphylococci holds a level of importance comparable to other critical clinical indicators in SAE risk. Early identification of this pathogen may therefore be instrumental in reducing SAE occurrence. Furthermore, antibiotic duration (HR=1.074, 95% CI: 1.056–1.092, P<0.001) was positively associated with SAE (**Table 2**; **Figure 2**).

**Table 2:** Multivariate logistic analysis of risk factors in patients with SAE.

Category	Reference	Training database		Testing database	
		HR (95%CI)	P	HR (95%CI)	P
Age, years	–	1.004 (1.000,1.007)	0.025	1.005 (1,1.01)	0.064
Gender	Male				
Female		1.185 (1.088,1.292)	<0.001	1.196 (1.051,1.361)	0.007
SOFA-24h	–	1.186 (1.166,1.206)	<0.001	1.17 (1.141,1.2)	<0.001
Charlson	–	0.987 (0.968,1.006)	<0.001	0.989 (0.960,1.018)	0.006
Platelets (g/dL)-min	–	1.003 (1.003,1.004)	<0.001	1.003 (1.002,1.003)	<0.001
White blood cell ( × 10^∧9^/L)-min	–	1.002 (0.996,1.007)	0.548	0.997 (0.989,1.005)	0.458
glucose_max	–	1.000 (0.999,1.000)	0.616	1 (1,1.001)	0.306
sodium_max	–	1.033 (1.023,1.043)	<0.001	1.045 (1.029,1.062)	<0.001
Length of admission to antibiotic use, days	–	1.074 (1.056,1.092)	<0.001	1.047 (1.024,1.071)	<0.001
Mechanical ventilation	No				
Yes		1.219 (1.116,1.333)	<0.001	1.204 (1.052,1.378)	0.007
Renal replacement therapy	No				
Yes		1.062 (0.843,1.337)	0.612	0.83 (0.591,1.166)	0.283
Microbiology type	Blood culture negative				
Staphylococcus aureus		1.435 (0.861,2.392)	0.166	1.86 (0.902,3.834)	0.093
Staphylococcus epidermidis		1.626 (0.654,4.044)	0.296	0.768 (0.253,2.329)	0.641
Thrombin negative Staphylococcus aureus		1.919 (1.331,2.769)	<0.001	2.316 (1.283,4.179)	0.005
streptococcus		0.522 (0.031,8.896)	0.653	0 (0,0)	1
Escherichia coli		0.831 (0.512,1.348)	0.453	1.314 (0.562,3.071)	0.528
klebsiella pneumoniae		1.410 (0.651,3.05)	0.383	0.409 (0.098,1.701)	0.219
Anaerobic bacteria such as fragile pseudomonas		1.407 (0.418,4.74)	0.582	0.522 (0.148,1.84)	0.312
Candida albicans		0.468 (0.135,1.623)	0.231	0.243 (0.019,3.071)	0.274
Grass green streptococcus		1.809 (0.666,4.916)	0.245	2.279 (0.272,19.09)	0.448
Methicillin resistant golden grape balls		4.722 (0.593,37.621)	0.143	523864868.535 (0,0)	0.999
Clostridium difficile		–	–	–	–
Mixed infection		0 (0,0)	0.999	0.886 (0.3,2.621)	0.827

### 3.3 Key influencing factors for GCS score in sepsis patients

The linear regression analysis revealed that SOFA score, platelet count, serum sodium level, mechanical ventilation, and renal replacement therapy significantly impact the GCS score in sepsis patients. SOFA score was inversely correlated with GCS (coefficient=-0.401, P<0.001), indicating that greater illness severity is linked to reduced neurological function. Similarly, increased platelet count and serum sodium were associated with lower GCS scores (platelet=-0.005, sodium=-0.062, both P<0.001). The need for mechanical ventilation (-1.298, P<0.001) and renal replacement therapy (-0.525, P=0.001) also correlated with lower GCS scores, reflecting greater neurological impairment in critically ill patients. The increased of length between admission and antibiotic use was associated with reduced GCS scores (-0.061, P<0.001). However, there is no significant correlation between different microbiology type and GCS (**Table 3**).

**Table 3:** Multivariate logistic analysis of risk factors in patients GCS.

Category	Reference	Training database		Testing database	
		HR (95%CI)	P	HR (95%CI)	P
Age, years	–	-0.005 (-0.01,0.001)	0.089	0.002 (-0.006,0.01)	0.610
Gender	Male				
Female		-0.093 (-0.224,0.037)	0.161	-0.091 (-0.29,0.109)	0.373
SOFA-24h	–	-0.401 (-0.423,-0.378)	<0.001	-0.376 (-0.41,-0.341)	<0.001
Charlson	–	0.073 (0.044,0.103)	<0.001	0.006 (-0.039,0.052)	0.780
Platelets (g/dL)	–	-0.005 (-0.005,-0.004)	<0.001	-0.004 (-0.005,-0.003)	<0.001
White blood cell ( × 10^∧9^/L)	–	0.002 (-0.006,0.01)	0.614	-0.002 (-0.014,0.011)	0.788
glucose_max	–	0 (0,0.001)	0.288	0 (-0.002,0.001)	0.744
sodium_max		-0.062 (-0.077,-0.047)	<0.001	-0.067 (-0.09,-0.043)	<0.001
Length between admission and antibiotic use, days	–	-0.061 (-0.077,-0.045)	<0.001	-0.037 (-0.061,-0.014)	0.002
Mechanical ventilation	No				
Yes		-1.298 (-1.437,-1.159)	<0.001	-1.177 (-1.39,-0.964)	<0.001
Renal replacement therapy	No				
Yes		-0.525 (-0.826,-0.224)	0.001	-0.751 (-1.222,-0.279)	0.002
Microbiology type	Blood culture negative	-0.036 (-0.081,0.01)	0.123	-0.019 (-0.095,0.057)	0.621

### 3.4 The impact of microbiological types on in-hospital mortality in SAE patients

Cox regression analysis of in-hospital mortality in patients with SAE revealed a significant association between coagulase-negative staphylococcal infection and a reduced risk of death compared to patients with negative blood cultures (HR: 0.596, 95% CI: 0.444–0.8, P=0.001). In addition to microbiology type, other factors such as SOFA score (HR=1.043, 95% CI: 1.027–1.06, P<0.001), mechanical ventilation (HR=0.821, P=0.003), and renal replacement therapy (HR=0.776, P=0.001) significantly impacted mortality risk (**Table 4**).

**Table 4:** Cox multivariable regres analysis of in-hospital mortality in SAE patients.

Category	Reference	SAE	
		HR (95%CI)	P
Age, years	–	1.007 (1.003,1.012)	0.002
Gender	Male		
Female		1.019 (0.917,1.132)	0.725
SOFA-24h	–	1.043 (1.027,1.06)	<0.001
Charlson	–	1.001 (0.979,1.022)	0.956
White blood cell ( × 10^∧9^/L)-min	–	1.001 (0.997,1.005)	0.532
glucose_max	–	1 (0.999,1)	0.436
sodium_max	–	1.002 (0.992,1.012)	0.697
Length of admission to antibiotic use, days	–	0.941 (0.932,0.951)	<0.001
Mechanical ventilation	No		
Yes		0.821 (0.72,0.936)	0.003
Renal replacement therapy	No		
Yes		0.776 (0.668,0.902)	0.001
Microbiology type	Blood culture negative		
Staphylococcus aureus		0.866 (0.620,1.209)	0.397
Staphylococcus epidermidis		0.900 (0.400,2.027)	0.800
Thrombin negative Staphylococcus aureus		0.596 (0.444,0.800)	0.001
streptococcus		–	–
Escherichia coli		0.577 (0.348,0.957)	0.033
klebsiella pneumoniae		0.747 (0.308,1.811)	0.519
Anaerobic bacteria such as fragile pseudomonas		0.420 (0.173,1.02)	0.055
Candida albicans		0.626 (0.2,1.961)	0.422
Grass green streptococcus		0.502 (0.283,0.89)	0.018
Methicillin resistant golden grape balls		3.423 (1.086,10.787)	0.036
Clostridium difficile		–	–
Mixed infection		0.899 (0.552,1.464)	0.668

## 4 Discussion

The present study aimed to elucidate the role of microbiological factors and clinical risk determinants in the development of sepsis-associated encephalopathy (SAE) and their influence on neurological function and in-hospital mortality. This study identified coagulase-negative Staphylococcus as a key microbial risk factor for sepsis-associated encephalopathy (SAE). Additionally, factors such as advanced age, female gender, higher Sequential Organ Failure Assessment (SOFA) scores, mechanical ventilation, and delayed antibiotic administration significantly contributed to the development of SAE. Notably, methicillin-resistant Staphylococcus aureus (MRSA) was associated with an increased in-hospital mortality rate among SAE patients.

Coagulase-negative staphylococci (CoNS) are the largest group of the Staphylococcus genus, mainly distinguished by the absence of one of the major staphylococcal virulence factors, the capacity to produce the coagulase enzyme. Coagulase-negative staphylococci includes over 40 species of Gram positive cocci (Becker et al., 2014). CoNS is the most representative bacteria in normal human skin and mucous membranes, which is a common pathogen on non-biological surfaces such as catheters and implants, especially in immunocompromised patients (Michels et al., 2021). CoNS accounted for 31% of all in-hospital bloodstream infections in a total of 49 hospitals in the United States over a period of 31 years (Wisplinghoff et al., 2004). This discovery has been confirmed in several other queues (Rosenthal et al., 2023). For example, an observational study in Germany evaluated the prevalence of nosocomial infections in a university hospital and found that CoNS was the second most common cause of nosocomial infections (Ott et al., 2013). Recent studies have revealed a close correlation between CoNS and the increased use of implantable medical devices such as heart valves or joint replacements (Noshak et al., 2020). The widespread use of antibiotics is related to the prevalence of CoNS, which accelerates the spread of resistance genes (Lopes et al., 2025). Due to its protective properties and its composition with polysaccharides and some proteins, the biofilm matrix prevents the penetration and action of antibiotics and their diffusion through the biofilm structure, exacerbating the increase in resistance and tolerance, making it difficult to treat and eliminate (Lebeaux et al., 2014). Moreover, CoNS are being increasingly studied within veterinary medicine, while their role as disease-causing pathogens in animals is still regarded as small. Although the relevance for human disease is not fully established, it has been shown that CoNS-inhabiting animals can display a wide range of antimicrobial resistances, and thus may potentially serve as a reservoir of resistance genes (Ruiz-Ripa et al., 2020). The drug resistance of CoNS makes it difficult to be treated with antibiotics and increases the inflammatory response time, which may be one of the reasons for the increased risk of septic encephalopathy in patients infected with CoNS. At present, the specific pathological mechanism is not clear and requires further exploration through basic research.

In addition, consistent with previous research findings, this study found that women, those with higher SOFA scores within 24 hours of admission, higher Charlson index, longer length of admission to antimicrobial use, and those receiving mechanical ventilation had a higher risk of SAEs. According to our research, SOFA score is a significant risk factor for SAE and has advantages in assessing the severity and prognosis of encephalopathy, consistent with previous reports (Zhang et al., 2024). Compared to non SAE patients, SAE patients have more severe conditions, manifested by higher costs, longer hospital stays, and longer duration of mechanical ventilation. It should be noted that a GCS score<15 is used to diagnose SAE patients; However, GCS score is a component of SOFA and APACHE II scores. Patients with higher SOFA scores are more likely to have SAEs, which may lead to biased conclusions (Kim et al., 2020). In this study, advanced age was a significant risk factor for SAE, with older patients having a higher risk of sepsis, while critically ill patients with underlying diseases typically progressed faster and had poorer prognosis. Especially if there is a high medical history, these patients may be more prone to central nervous system complications (Sonneville et al., 2015; Jin et al., 2022).

There are several limitations in the study. First, the definition of SAE was based on the retrospective analysis. Lack of imaging data may cause SAE’s cohort to expand. This comes from the limitations of retrospective studies, where the quality of data sources varies and the timeliness and completeness of data are difficult to control.

Second, The blood culture results may be due to contamination, but studies have shown that if clinical symptoms indicate infection, a positive blood culture is sufficient for diagnosis (Favre et al., 2005). It has clinical significance, especially in cases of high mortality clinical syndromes such as sepsis. All patients in this study were screened according to Sepsis 3.0 criteria and exhibited signs and symptoms of systemic infection. In cases of true bloodstream infection, Staphylococcus epidermidis typically demonstrates faster growth and higher bacterial load. In this study, blood cultures became positive within 72 hours, suggesting a high likelihood of hematogenous infection. Third, the condition of critically ill patients is critical and complex, and many confounding factors cannot be ruled out. And the data for this study is sourced from The MIMIC-IV database, and the applicability of the results needs to be validated in other races or countries. Finally, this study did not include all pathogens identified in the blood culture results. Only 12 major blood-borne pathogens were selected, with the minimum number of cases being 2 patients. Due to the wide variety of pathogens and the small number of patients with pathogens not included in the analysis (ranging from 0 to 2), this exclusion does not affect the final conclusion. In the future, the sample size can be expanded to verify the impact of small sample bacteria on SAE.

## 5 Conclusion

This study identified coagulase-negative Staphylococcus as a key microbial risk factor for SAE. Additionally, advanced age, female gender, higher SOFA scores, mechanical ventilation, and delayed antibiotic administration significantly contribute to the development of SAE. Furthermore, methicillin-resistant Staphylococcus aureus (MRSA) is associated with an increased in-hospital mortality rate among SAE patients. Implementing routine monitoring programs for patients with the above risk factors can reduce the burden of SAE related diseases.

## Data Availability

The raw data supporting the conclusions of this article will be made available by the authors, without undue reservation.
