# Supplementary material for: Microbiological and clinical predictors of sepsis-associated encephalopathy in bloodstream infections: a retrospective cohort study
Source: Front Cell Infect Microbiol. 2025 Mar 7;15:1548370. doi: 10.3389/fcimb.2025.1548370 (PMC11925888; doi:10.3389/fcimb.2025.1548370)
Supplement: Supplementary file 1 [file Table1.docx]

Supplemental table 1. Comparison between training level and validation set.

| Category | Training database | Testing database | *P* value |
| --- | --- | --- | --- |
| Age, median (IQR) | 66.85(16.06) | 67.18(15.96) | 0.243 |
| **Gender,n (%)** |  |  | 0.836 |
| Male | 6590(58.3) | 2835(58.5) |  |
| Female | 4706(41.7) | 2010(41.5) |  |
| **Coexisting illness , n (%)** |  |  |  |
| Charlson | 4(3-7) | 5(3-7) | 0.202 |
| Hypertension | 4802(42.5) | 2080(42.9) | 0.621 |
| Diabetes | 3227(28.6) | 1377(28.4) | 0.850 |
| Respiration | 3033(26.9) | 1297(26.8) | 0.916 |
| Cardiovascular | 2046(18.1) | 885(18.3) | 0.816 |
| Renal | 2381(21.1) | 1037(21.4) | 0.643 |
| **Microbiology type, n (%)** |  |  | 0.401 |
| Culture-negative | 10691(94.6) | 4610(95.1) |  |
| Staphylococcus aureus | 100(0.9) | 49(1.0) |  |
| Staphylococcus epidermidis | 34(0.3) | 16(0.3) |  |
| Thrombin negative Staphylococcus aureus | 229(2.0) | 89(1.8) |  |
| enterococcus |  |  |  |
| streptococcus | 2(0) | 1(0) |  |
| Escherichia coli | 88(0.8) | 29(0.6) |  |
| klebsiella pneumoniae | 40(0.4) | 9(0.2) |  |
| Anaerobic bacteria such as fragile pseudomonas | 15(0.1) | 12(0.1) |  |
| Candida albicans | 11(0.1) | 3(0.1) |  |
| Grass green streptococcus | 28(0.2) | 8(0.2) |  |
| Methicillin resistant golden grape balls | 14(0.1) | 3(0.1) |  |
| Baumann Acinetobacter baumannii MIX |  |  |  |
| Clostridium difficile | 2(0.01) | 0(0) |  |
| Baumann Acinetobacter baumannii |  |  |  |
| Mixed infection | 42(0.4) | 16(0.3) |  |
| **Organ replacement therapy , n (%)** | |  |  |
| Mechanical ventilation | 6935(61.4) | 3024(62.4) | 0.221 |
| Renal replacement therapy | 602(5.3) | 253(5.2) | 0.780 |
| **Score system, median (IQR)** |  |  |  |
| SAPS II | 38(30-48) | 38(31-47) | 0.834 |
| SOFA-24h | 5(3-8) | 5(3-7) | 0.403 |
| GCS | 14(10-15) | 14(11-15) | 0.153 |
| BMI | 29.32(7.69) | 29.22(7.86) | 0.526 |
| Use of vasopressors [n (%)] | 5956(52.7) | 2524(52.1) | 0.461 |
| Length of admission to antibiotic use, days, mean(SD) | 1.84(4.04) | 1.87(4.14) | 0.763 |
| Length of ICU, days, mean(SD) | 4.81(4.89) | 4.74(4.74) | 0.438 |
